# Fetal Alcohol Spectrum Disorder (FASD) Associated Neural Defects: Complex Mechanisms and Potential Therapeutic Targets

**DOI:** 10.3390/brainsci3020964

**Published:** 2013-06-19

**Authors:** Pooja Muralidharan, Swapnalee Sarmah, Feng C. Zhou, James A. Marrs

**Affiliations:** 1 Department of Biology, Indiana University-Purdue University Indianapolis, Indianapolis, IN 46202, USA; E-Mails: pmuralid@iupui.edu (P.M.); ssarmah@iupui.edu (S.S.); 2 Department of Anatomy and Cell Biology, Stark Neuroscience Research Institute, Indiana University School of Medicine, Indianapolis, IN 46202, USA; E-Mail: imce100@iupui.edu

**Keywords:** fetal alcohol syndrome, fetal alcohol spectrum disorder, ethanol, central nervous system, cranial neural crest cells, vitamin deficiency

## Abstract

Fetal alcohol spectrum disorder (FASD), caused by prenatal alcohol exposure, can result in craniofacial dysmorphism, cognitive impairment, sensory and motor disabilities among other defects. FASD incidences are as high as 2% to 5 % children born in the US, and prevalence is higher in low socioeconomic populations. Despite various mechanisms being proposed to explain the etiology of FASD, the molecular targets of ethanol toxicity during development are unknown. Proposed mechanisms include cell death, cell signaling defects and gene expression changes. More recently, the involvement of several other molecular pathways was explored, including non-coding RNA, epigenetic changes and specific vitamin deficiencies. These various pathways may interact, producing a wide spectrum of consequences. Detailed understanding of these various pathways and their interactions will facilitate the therapeutic target identification, leading to new clinical intervention, which may reduce the incidence and severity of these highly prevalent preventable birth defects. This review discusses manifestations of alcohol exposure on the developing central nervous system, including the neural crest cells and sensory neural placodes, focusing on molecular neurodevelopmental pathways as possible therapeutic targets for prevention or protection.

## 1. Introduction

Fetal alcohol syndrome (FAS), which affects approximately 2 to 7 of 1000 live births, is a severe developmental deficit caused by maternal ingestion of ethanol during pregnancy. Prevalence of FAS is variable for different populations depending on the maternal alcohol consumption rate in the given population. Affected individuals exhibit a spectrum of defects including specific physical, cognitive, psychomotor, behavioral, and learning disabilities with identifiable facial dysmorphology. However, many more affected subjects are not readily identifiable by the facial features. This previously undiagnosed population is estimated as high as ten times that of FAS and is now categorized as fetal alcohol spectrum disorders (FASD) [1]. Affected individuals suffer lifelong disabilities and may require long-term support, and so, FASD has a huge economic and societal impact. A significant amount of research work was done since FAS was defined in 1973 to understand alcohol’s teratogenic effect during development. However, it is still not clear how alcohol consumption produces a wide spectrum of physical and mental disabilities. This may be due to differences in dosages, timing of alcohol exposure (stage-dependent and/or duration dependent) or differences in comorbid environmental and genetic factors. For example, some effects in FAS are greater or characteristically different when alcohol exposure is earlier (first trimester) than later during pregnancy, and experimental evidence in animal models supports these findings. This review discusses the alcohol-induced nervous system defects, including central nervous system, neural placodes and cranial neural crest cells. Progress on understanding mechanisms underlying neural teratogenesis is reviewed. 

### 1.1. Alcohol Induced Craniofacial Defects

Maternal ethanol exposure, in severe cases, significantly alters craniofacial features of an individual. Key diagnostic anomalies include small head circumference, flat midface, short nose, indistinct philtrum, low nasal bridge, thin upper lip, micrognathia, short palpebral fissures and other defects [2,3]. Studies conducted in different animal models, particularly in mice, that recapitulate a similar spectrum of brain and facial malformations upon alcohol exposure provided strong evidence that alcohol induced these craniofacial alterations [4,5,6,7,8]. Pioneering work by Webster and co-workers demonstrated that alcohol exposure in mice on gestation day (GD) 7 or 8 was sufficient to produce severe facial malformations in offspring [4]. Subsequent research was directed towards understanding vulnerability of facial structures in developing embryos to alcohol exposure [5,7,9]. The FAS mouse model recapitulates craniofacial defects [5,7,10] and was shown to be remarkably similar to those of affected humans [2,3]. It was shown that a critical developmental window for alcohol-induced alteration of facial development comprised gastrulation through neurulation during embryogenesis [6]. The fact that alcohol produced craniofacial malformations in mice resembling those in human FAS patients gave investigators confidence to study alcohol’s teratogenic effects in detail using animal models other than rodent, like chick, *Xenopus* and zebrafish [8,11,12].

### 1.2. Alcohol Induced Central Nervous System Development Defects

The key consequences of prenatal alcohol exposure are defects in central nervous system development. Although reduced growth and specific minor facial malformations are most characteristic of FAS, the effects of alcohol on brain development are most significant because they lead to long-term cognitive and behavioral deficits in FAS and FASD patients. Earlier studies used autopsy to understand structural brain defects associated with prenatal alcohol exposure. Consistent abnormalities reported in different autopsy studies were microcephaly (small head) and microencephaly (small brain) [13]. Autopsies also reported various other abnormalities, such as cases of agenesis or malformation of the corpus callosum; small, poorly formed cerebellum; alobar and semilobar holoprosencephaly; hydrocephalus; and ventriculomegaly [14,15]. One of the mechanisms leading to these brain defects is believed to stem from errors in migration of neuronal and glial cells [13]. Various autopsy reports demonstrated extreme variability in the nature and degree of brain malformations in children with FAS, which led these investigators to think that there might be no specific pattern of neurological deficits attributable to FAS [13]. However, autopsies were done on limited number of fetuses, neonates or small children, and often, these individuals represented the most severe cases of FAS. 

The devastating influence of alcohol on brain development in less severely affected individuals remained unappreciated for several years. Fortunately, recent advances in medical imaging technology with novel computational brain image analysis techniques has transformed FASD research, allowing visualization of brain structures in living FASD individuals. Magnetic resonance imaging (MRI) gives the opportunity to study affected living individuals exhibiting physical and functional deficits across varying levels of severity in a non-invasive way. These studies provided new insights into the brain defects caused by prenatal alcohol exposure. MRI is also advantageous because it allows larger study sample size (than autopsy studies, for example), helping investigators do quantitative analysis, identify consistent findings, and examine the specific nature of alcohol’s effect on the brain. To date, numerous MRI studies were done on FASD patients, and those reports were extensively reviewed [15]. Similar to autopsy studies, MRI studies consistently demonstrated total brain volume reduction in patients [15]. In addition, it was frequently reported that prenatal alcohol exposed patients had reduced size in cerebrum, cerebellum, hippocampus, and basal ganglia (including caudate nuclei), and corpus callosum malformations. Moreover, MRI studies showed that although brain structural abnormalities are widespread, all brain structures were not affected equally. Studies that measured occipital lobe reported no significant changes in volume, shape and displacement. However, frontal, parietal, and temporal lobes showed significant changes: increased cortical thickness; decreased white and grey matter volumes; increased grey matter density; and other histological changes were associated with cerebral lobe defects in subjects prenatally exposed to alcohol [15]. More recently, developmental cortical thinning [16], abnormal cortical thickness alterations [17], and reduced callosal thickness and area specifically in the anterior third and the splenium, [18] were reported in FASD. 

Human FASD associated brain phenotypes were consistently recapitulated in the rodent central nervous system following prenatal ethanol exposure [5,7,19,20,21]. Studies on animal models showed that ethanol interferes in all stages of brain development, but exposure at various developmental stages caused distinct brain developmental abnormalities. Sulik and colleagues showed unique patterns of regional brain abnormalities resulted from stage-specific ethanol exposure between GD7 and GD10 in mice [9,19,21,22]. At the cellular level, ethanol exposure was reported to cause a permanent loss of neurons; ectopic neuron formation; and altered synaptogenesis and myelinogenesis. Studies showed that within a given brain region, different neuronal populations exhibit different degrees of vulnerability to alcohol. In the cerebellum, Purkinje cells were reported to be more susceptible to alcohol exposure than other cell types [23,24,25], and among the Purkinje cells, those at the stage of extending dendrites were more susceptible to alcohol than more mature Purkinje cells, at later stages of differentiation [26].

Early work on alcohol-induced CNS defects focused on mechanisms underlying learning disabilities examined hyperactivation of GABA_A_ receptors that triggers a neurodegenerative response, which is particularly sensitive during the period of synaptogenesis (in humans, this occurs during the last weeks of pregnancy and early postnatal development). Experiments on mouse embryos showed massive ethanol-induced degeneration of neurons in many parts of the brain that may play a role in learning, memory, sensory information processing and cognitive defects. These studies primarily hypothesized that neurodegenerative response to ethanol exposure could be attributed to GABA mimetic properties of ethanol [27].

**Figure 1:**
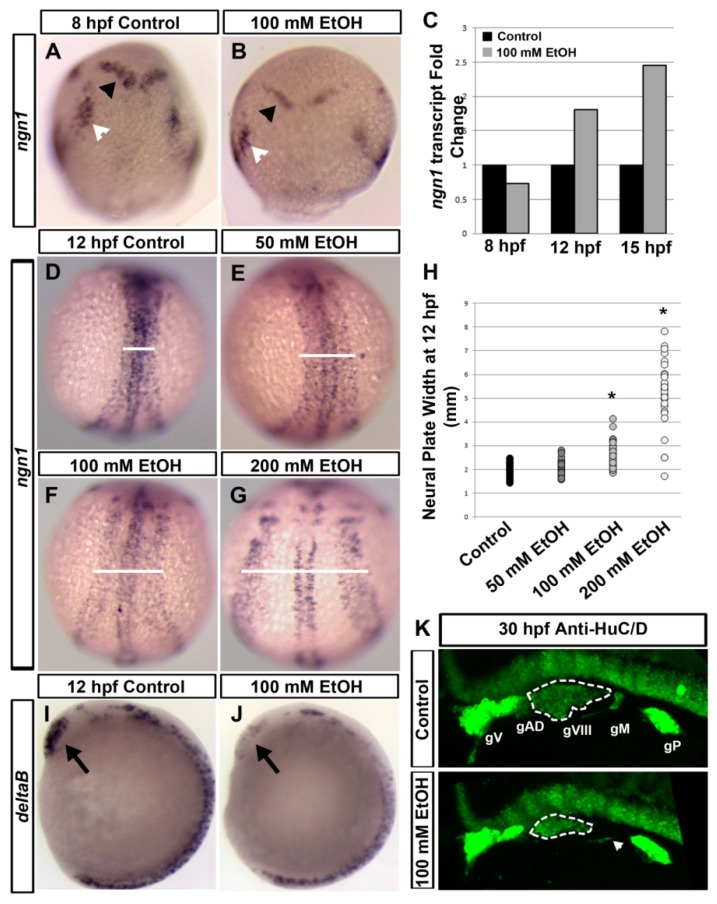
Ethanol-induced neural defects in zebrafish embryos. Zebrafish embryos were exposed to ethanol for various times during gastrulation and somitogenesis stages. (**A**,**B**) Ethanol treated and control embryos were stained by in situ hybridization (ISH) to detect proneural gene, *neurogenin1* (*ngn1*) expression patterns at 8 hpf. Reduced *ngn1* expression was seen after 100 mM ethanol treatment. Black arrowhead indicates presumptive midbrain region. White arrowhead indicates trigeminal ganglion. Dorsal view, anterior to the top. (**C**) Quantitative-PCR detecting *ngn1* transcripts at 8, 12 and 15 hpf showed that ethanol reduced *ngn1* expression at 8 hpf, and then, increased *ngn1* expression at 12 and 15 hpf, as compared to control levels. Ribosomal protein *rsp15* gene was used as an endogenous control. (**D**–**G**) *ngn1* ISH at 12 hpf with increasing concentrations of ethanol (50–200 mM) showed neural convergence defects. White line indicates the neural plate width, which is wider after ethanol treatment. Dorsal view, anterior to the top. (**H**) Quantification of neural plate width demonstrates neural convergence defect. The scatter plot shows an increasing neural plate width after ethanol treatment in a dose-dependent manner. Asterisk indicates a statistically significant difference (*p* < 0.0001) from the control embryos using Student’s *t*-test. The position of neural tube width measurement was at the central point of the neural tube. (**I**,**J**) Ethanol reduced deltaB expression in somitogenesis stage embryos. ISH on embryos using Notch ligand, *deltaB* probe showed considerable reduction in *deltaB* expression in the forebrain due to ethanol exposure. Black arrow indicates forebrain. Lateral view, anterior to the top. (**K**) Ethanol reduces cranial ganglia development. Anti-HuC/D immunostaining of embryos at 30 hpf labels the mature differentiated neurons. A reduction in the volume of cranial ganglia was seen after ethanol treatment. White dashed lines marks gVIII; White arrowhead indicates defective gM. Abbreviations: gV, trigeminal; gAD anterodorsal ganglion; gVIII, statoacoustic ganglion; gM, middle lateral line ganglion; gP, posterior lateral line ganglion.

Animal models and tissue-culture studies identified numerous potential mechanisms of alcohol induced craniofacial and brain defects. Previous experiments showed higher sensitivity during gastrulation stages to environmental toxin-induced birth defects, including alcohol exposure [9]. Experiments on zebrafish embryos treated with ethanol during gastrulation and somitogenesis showed changes in critical signaling molecule and transcription factor expression, including the notch ligand, *deltaB*, and the proneural gene, *neurogenin1* (*ngn1*) (Figure 1A–J). Expression changes in *ngn1* indicate that neural specification and differentiation was delayed, including brain, spinal cord and cranial ganglia progenitors (Figure 1A,B). Expression of *ngn1* rebounded and overshot the control levels at later developmental times (Figure 1C). Neural tissue convergence was also delayed, showing a wider neural plate after ethanol exposure in a dose-dependent manner (Figure 1D–G). Defects in convergence and extension cell movements during gastrulation were shown to be caused by ethanol exposure [28]. Reduced neural specification and differentiation could produce defects in specific neural structures. Cranial ganglia were examined in 30 hpf (hours post-fertilization) zebrafish after ethanol exposure using HuC/D antibody staining, which detects differentiated neurons. Cranial ganglia showed significant reduction in volume after ethanol exposure as compared to untreated controls (Figure 1K). This suggests that defects in cell movement and signaling during early stages of development may lead to more severe defects at later stages. For example, the 8th ganglion (gVIII) was reduced, which could lead to auditory nerve defects, as seen in FASD patients (See below).

### 1.3. Ethanol Induced Defects in Sensory System Development

Effects on sensory systems are among the critical neural defects that can lead to abnormalities in a child’s social development. Sensory defects are frequently associated with FASD, including auditory, visual and language deficits.

#### 1.3.1. Auditory Defects

Epidemiological studies showed a close association between craniofacial dysmorphism and hearing deficits. These studies revealed that 83% of children with FAS had conductive hearing loss and 28% demonstrated sensorineural hearing loss [29]. Auditory system, comprising of the outer, middle and the inner ear, has a dual origin of the thickened ectodermal placode and cranial neural crest cells (CNCC). The conductive hearing loss occurs because of problems in sound conduction by the outer or middle ear, and can be caused by ear infections, blockage/decreased motion of the ear ossicles or tympanic membrane. Sensorineural hearing loss occurs due to abnormalities or loss of sensory hair cells in the cochlea. Increased rates of recurrent middle ear infections potentially due to Eustachian tube abnormalities were also associated with craniofacial defects in children with FAS, which was reviewed with regards to its role in language and cognition by Cone-Wesson [30]. 

Most information regarding ethanol-induced sensorineural hearing loss comes from studies on mouse embryos, which showed high sensitivity of otic primordium development to ethanol exposure. Increased cell death at the rostral border of the invaginating otic placode was observed in histological sections [31]. These cells later form the functional sensory cells in the inner ear and neurons of the eighth cranial nerve. Loss of these cells could cause sensorineural hearing loss at later stages.

Pioneering studies by Church, M.W., 1987 [29], analyzed effects of prenatal ethanol exposure on auditory function, using auditory brainstem response as a measure. He found that embryonic ethanol exposure delayed maturation of the auditory system, and a significant number of ethanol exposed neonatal mice showed sensorineural hearing loss. A more recent study used the rat model to show maternal alcohol consumption had a dose-dependent effect on electroencephalograms obtained from auditory cortex in ethanol-exposed neonatal rats following acoustic stimulation [32]. 

Being at risk for auditory deficits coupled with CNS defects may contribute to problems with speech, language and auditory learning. However, a huge gap exists in our understanding of the mechanisms underlying the auditory manifestations of FASD. Additional studies are needed to characterize critical ethanol-sensitive events in otic placode and sensory development. A potential mechanism could include defective and delayed neuroectoderm development. These defects, combined with craniofacial abnormalities, could affect normal cell and tissue interactions during various developmental events, resulting in abnormal auditory function. For example, ethanol-induced CNCC defects will also need to be studied in order to completely understand otic vesicle defects.

#### 1.3.2. Olfactory Defects

Fetal alcohol exposure affects various neurodevelopmental processes, including olfactory development and function. Previous human epidemiological studies showed an increased risk for adolescent ethanol abuse was associated with fetal alcohol exposure. Studies by Youngentob and colleagues using adolescent rats showed an enhanced chemosensory acceptability for ethanol in individuals with prenatal ethanol exposure. Their study using rats showed that prenatal ethanol exposure produced an enhanced olfactory preference for ethanol odor and an increased ethanol intake [33,34]. However, factors underlying the enhanced ethanol orosensory (taste) and/or olfactory (smell) acceptability after fetal alcohol exposure are not yet identified. 

**Figure 2:**
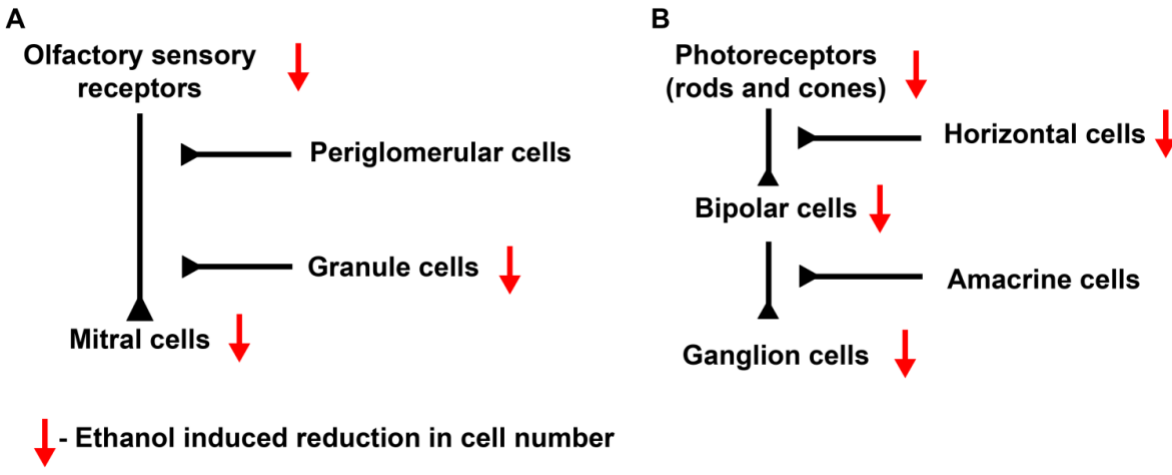
Effects of ethanol on olfactory and optic neuroplacodes. (**A**) Under normal conditions, olfactory sensory receptors synapse on to mitral/tufted cells in the glomeruli. This synapse can be modulated by two types of interneurons, namely periglomerular and granule cells. Studies have shown a reduction in the number of olfactory sensory neurons (OSNs), M/T cells and granule cells after ethanol treatment. (**B**) Normal development of the optic system produces various cell types in the retina. Photoreceptors synapse on bipolar cells, which then synapse on retinal ganglion cells. This circuit is modulated by interneurons, namely horizontal and amacrine cells. Ethanol reduced the number of rod photoreceptors, bipolar and ganglion cells. Horizontal cell dendrite arborization was also reduced by ethanol exposure. Black schematic synapses indicate a simplified synapse circuit. Red arrows indicate effects of ethanol on cell number.

The olfactory bulb is a laminated structure consisting of olfactory sensory neurons (OSNs), which synapse with dendrites of the glutamatergic mitral and tufted cells (M/T cells) in olfactory bulb glomeruli. The M/T cells comprise a majority of the olfactory tract. M/T cells are also modulated by inhibitory GABAergic interneurons, periglomerular and granule cells (Figure 2A). Early histological analysis of the rat neonate olfactory bulb showed a significant decrease in cell number in the M/T cells and granule cell layers (27.9% and 26.4% respectively) after ethanol exposure (Figure 2A) [35]. More recently, advanced high-resolution MRI scans of mouse brains showed that the region with greatest proportional decrease in volume after alcohol exposure was the olfactory bulb [21,36]. Odor discrimination between highly similar odors was also impaired in prenatal alcohol exposed mice [36].

To identify the olfactory developmental mechanisms disrupted by fetal alcohol exposure in more detail [37], gene chip experiments showed reduced expression of the metabotropic glutamate receptor 2 (mGluR2) gene which are expressed by the OSNs and M/T cells. Gamma-aminobutyric acid (GABA) B receptor 1 (GABA B1) gene expression was also decreased as a consequence of fetal alcohol exposure. Adrenergic receptor 1 (Adra1, highest expression density in the olfactory bulb in the brain) expression was also reduced. Adra1 plays an important role in signal enhancement in OSNs. In addition to these neurotransmitter receptor genes, expression of many other genes involved in synaptic transmission, development and cell fate specification were affected by ethanol exposure. Their studies suggest that alcohol induced defects are two-fold: reducing number of specific cells in the olfactory bulb; and interrupting normal neurotransmission processes. Additional characterization of the olfactory bulb developmental defects are needed to understand alcohol-induced olfactory system defects, including systematic analysis of gene expression changes and cell death in the developing olfactory bulb.

#### 1.3.3. Ocular Defects

FAS is associated with visual system developmental defects. Epidemiological studies showed association of FAS with defects in almost all parts of the eye. Severe cases had microphthalmia, coloboma and cataract. Milder defects were also seen in FAS/FASD patients, including strabismus, high refractive errors and reduced visual acuity. Ninety six percent of children suffering from FASD showed ophthalmological defects [38]. Human epidemiological studies showed persistently poor vision that remained unchanged even in follow-up measurements. The children with FAS also frequently display optic nerve hypoplasia and abnormal electroretinograms.

The eye is an extremely sensitive indicator of prenatal abnormalities induced by teratogen exposure, and assessment of ocular defects is relatively easy, clinically accessible and well defined. Retinal development is well characterized in most animal models. Following evagination from diencephalon, retinal developmental signaling and cell specification events are initiated by inductive signals from the optic stalk. Interactions with the ectoderm (lens placode) and the perioptic mesenchyme (derived from CNCCs) help orchestrate eye development. Specific signaling events propagate the neurogenic wave of differentiation, and lamination of retinal cell types occurs as the tissue matures. Photosensory receptors of the retina (the rods and cones) synapse on interneurons, namely bipolar and horizontal cells (Figure 2B). These neurons then synapse on amacrine and ganglion cells. The retinal ganglion cells form the optic nerve and project their axons to retinorecipient brain regions for further processing of the visual stimuli (Figure 2B). Müller glial cells are the astroglia of the retina. These cells not only maintain ionic homeostasis but may also represent a stem cell population within the retina.

Initial studies using various vertebrate animal models documented significant reduction in ganglion cell number and changes in optic nerve axon structure, specifically showing delayed or defective myelination following ethanol exposure [38]. This also correlated with defective or delayed gliogenesis seen in embryos after ethanol exposure. Subsequent studies dissected mechanisms underlying ethanol-induced retinal defects. Clinical examinations identified that scotopic electroretinograms (ERGs) showed more deficiencies than photopic ERG [39]. Experimental evidence supporting this finding showed reduced rhodopsin expression in mouse embryos exposed to ethanol [40]. Effects of ethanol on photoreceptors and their ERGs were dose-dependent [41]. Using zebrafish as a model, Bilotta *et al*. [42], showed that a significant reduction in eye diameter and optomotor response in fish treated with ethanol during somitogenesis (12–24 hpf). This developmental period includes optic primordium specification and neurogenesis events. Treatment during time windows before or after 12–24 hpf did not cause as severe defects as those produced by treatment during 12–24 hpf. Also, higher ethanol concentrations during this sensitive period induced severe lamination defects and cyclopia [43]. The authors also suggested that retinal defects may be caused by impaired migration of prechordal plate during retinal morphogenesis because mutants showing similar eye defects exhibit prechordal plate migration defects [8]. Experiments on *Xenopus* embryos showed that ethanol exposure during early development caused abnormal expression of eye morphogenetic genes such as *Pax6* and *Tbx3*, which could result from increased in sonic hedgehog (*shh*) expression levels [44].

Studies on zebrafish showed that the presence of ethanol during retinal neurogenesis (24–48 hpf) induced persistent microphthalmia caused by increased cell death in the retina and lens as compared to untreated embryos [45]. A significant reduction in the number of each retinal cell types including photoreceptors and ganglion cells was seen (Figure 2B). The investigators designed rescue experiments to target signaling pathways that may be affected by ethanol, including *shh* and retinoic acid (RA) signaling. Their experiments showed that ethanol mediated defects were not rescued by these treatments, suggesting that retinal defects were caused by alternative pathways during this developmental period. However, photoreceptor differentiation could be rescued by RA treatment.

Transmission of visual signals from photoreceptors occurs via the interneurons, namely the bipolar and horizontal cells. Studies on mouse embryos showed significant, persistent and dose-dependent reduction in the number of bipolar cells (PKC-α positive) [46]. Other experiments showed significant reduction in the dendritic receptive field of horizontal cells. However, since bipolar and horizontal cells are among the last cell types to differentiate and cell types that continue differentiation postnatally, defects in bipolar and horizontal cells may be secondary effects of earlier defects in the retina. To summarize, ethanol induced defects on retinal development are dose and time dependent, leading to a severe, persistent defects in the structure and function of all retinal cell types. Additional experiments are necessary to understand specific signaling mechanisms underlying retina and lens defects.

Various cell types that comprise the above-described sensory systems show severe reduction in numbers and functional defects after ethanol exposure. Ethanol-induced defects on the precursor cells of the sensory systems, including the placode and CNCCs, are not clearly understood. Systematic examination of developmental defects in sensory systems and evaluating mechanisms underlying these defects, such as delayed or defective signaling between the placode and neuroectodermal tissues, and CNCC cell death, will be insightful in understanding associated psychomotor defects in FASD patients and to evaluate therapeutic interventions.

### 1.4. Ethanol and Neural Crest Cells

Studies using mouse and chick showed that alcohol exposure at specific stages of early embryonic development did not equally affect all cells within the embryo. Alcohol administration during gastrulation and neurulation produced a major insult to the anterior neural plate, and in particular, the population of cells destined to give rise to brain and facial structures, CNCC [6,47,48,49,50]. These cells originate from the dorsal neural tube following its closure and migrate to specific locations [51]. Neural crest cells differentiate and form diverse structures, including majority of the head connective and skeletal structures; nerves; pigment cells; aorticopulmonary septum and conotruncal cushions of heart; enteric ganglia; and sensory and sympathetic ganglia [52]. CNCC also contribute to various parts of sensory organs, such as cartilage in the middle ear and periocular mesenchyme surrounding the retina, which later forms the corneal epithelium, ciliary body and sclera. CNCCs give rise to specific features that are affected in FAS patients. Studies of alcohol induced cell death have been extensively reviewed [50]. Those findings suggested that prenatal alcohol exposure induced apoptotic elimination of premigratory and migratory neural crest cells. Alcohol exposure at distinct developmental window differentially affected the CNCC death [6,47,50]. Researchers have extensively investigated mechanisms that could trigger the CNCC death.

## 2. Potential Mechanisms Underlying Ethanol-Induced Neural Defects: Possible Therapeutic Targets

Numerous molecular mechanisms underlying ethanol-induced defects in human patients were hypothesized and studied experimentally in rodents and other vertebrates. It is likely that ethanol toxicity for the embryo proceeds by more than one mechanism, and primary targets and secondary effects of ethanol toxicity still need to be identified.

### 2.1. Ethanol-Induced Nutritional Deficiencies

Vitamin deficiencies were linked to a number of diseases, which are particularly prevalent in populations of low socioeconomic conditions. Alcohol consumption also aggravates malnutrition by interfering with the absorption, digestion and utilization of nutrients consumed. Chronic alcoholics tend to have a poor nutritional status and vitamin deficiencies, including vitamins A, B-complex, E and folic acid [53]. Thus, a potential contributing factor causing an increased FASD incidence in lower socioeconomic populations is severe nutritional deficiency, aggravated further by chronic alcohol consumption.

#### 2.1.1. Retinoic Acid

Retinoic acid (RA) is a morphogen, which plays a critical role in the vertebrate nervous system and limb morphogenesis. RA gradients are critical in the establishment of anterior posterior axis of the nervous system [54]. Vitamin A (retinol) is oxidized to RA in a developing embryo. The RA morphogen gradient is tightly controlled in specific tissues by regulating gene expression of alcohol/aldehyde dehydrogenases (ADH/ALDHs) and various RA degradation enzymes. RA is membrane permeable. It binds nuclear retinoic acid receptors and activates transcription of specific genes in target tissues. Reduced RA signaling due to mutations in RA synthesizing enzymes or due to vitamin A-deficient diets during pregnancy can produce complex developmental phenotypes that resemble FAS-associated defects.

Enzyme kinetics of isolated human liver ADHs showed that ethanol competitively inhibits retinol oxidation [55]. Presence of ethanol during embryonic development may reduce RA in target tissues due to the competition between ethanol detoxification and vitamin A metabolism. The resulting deficit in RA signaling may produce embryonic malformations. Many studies have examined the inhibition of RA synthesis as a potential mechanism underlying the FASD phenotype [56,57]. RA rescue experiments were also used to test its role in ethanol-induced defects during gastrulation, ocular and limb development.

**Figure 3:**
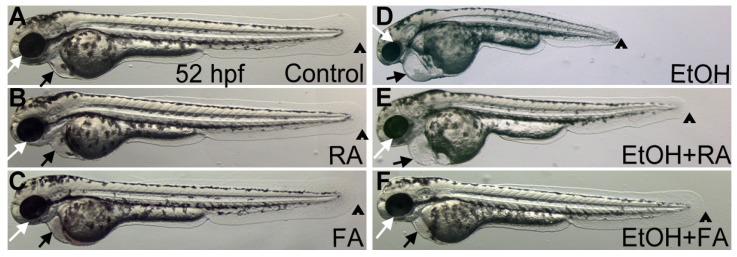
Retinoic acid or folic acid supplementation with ethanol during zebrafish embryogenesis significantly rescues ethanol induced morphological defects. (**A**–**C**) Lateral view of live images showed normal embryonic morphology in the control, 1 nM RA and 75 μM FA treated embryos at 52 hpf. (**D**) Ethanol treated (150 mM) embryos exhibited shorter body, small eye, and heart edema at 52 hpf. (**E**) Ethanol (150 mM) and RA (1 nM) co-treated embryo showed rescue of ethanol-induced small eye and short body length defect, but not the pericardial edema. (**F**) Ethanol (150 mM) and FA (75 μM) co-supplementation rescued most of ethanol-induced defects including shorter body, small eye, and heart edema. Ethanol plus FA supplemented embryo (**F**) showed normal morphology at 52 hpf. Black arrow indicates heart; white arrow indicates eye; arrowhead indicates posterior end of the embryo. Based on [61].

Studies on *Xenopus* and zebrafish embryos showed dramatic rescue of ethanol-induced phenotypes by supplementing with low concentrations of various retinoids [58,59,60]. RA rescued ethanol-induced effects, including anterior-posterior neural axis length, craniofacial and ocular abnormalities. Experiments using *Xenopus* embryos showed a strong similarity between the defects induced by high ethanol concentrations and those induced by combined treatment of low ethanol concentration and RA signaling inhibitor such as DEAB or retinaldehyde dehydrogenase (RALDH) knockdown. RA-responsive *hox* gene expression was suppressed at similar levels using either high ethanol concentration or low ethanol concentration plus RA signaling inhibitor [59], suggesting that activation of RALDH activity can compensate, in part, for the deleterious effect of ethanol. Rescue experiments using low concentrations of RA or RALDH overexpression could restore most ethanol-induced defects. Using zebrafish, Marrs *et al*. [57], showed that ethanol exposure during gastrulation and somitogenesis caused reduced epiboly, short body length and craniofacial defects (Figure 3D). RA supplementation (1 nM) rescued most of these defects. However, RA plus ethanol treated embryos showed cardiac edema (Figure 3E, black arrow). These experiments strongly suggested that many but not all developmental malformations observed in FASD were caused by reduced concentrations of RA during embryonic development.

However, reports by Kane *et al.* [61] indicate elevated physiological RA levels after ethanol exposure at multiple loci in the embryonic and adult mouse tissues, particularly brain tissues. These experiments suggest that tissue-specific increases in RA may contribute to the etiology of ethanol-induced defects. In another study, mechanisms underlying ethanol-induced retinal defects were dissected. They found that alcohol treatment during retinal neurogenesis (24–48 hpf) produced ocular manifestations, including defective retinal ganglion cell and photoreceptor differentiation. RA rescued photoreceptor differentiation, but did not rescue microphthalmia or ganglion cell defects [62].

The use of RA as a therapeutic is limited because it is a known teratogen. Overexposure to RA can disrupt the morphogen gradient and cause embryonic malformations. From a therapeutic perspective, a safer and more effective agent is needed to prevent or reverse potential ethanol-induced defects.

#### 2.1.2. Folic Acid

Folate has several important roles in multiple metabolic pathways. It is an essential cofactor in nucleic acid synthesis. Conversion to tetrahydrofolate (THF) involved in one carbon metabolism, is an important pathway leading to epigenetic changes by the transfer of methyl groups onto DNA and histones, which regulates gene expression. Folate also plays an important role as an antioxidant. Its deficiency during pregnancy may cause neural tube closure defects and other severe fetal malformations. Supplementation of FA in pregnant mothers lowers the rate of neural tube closure defects, congenital heart defects and cleft lip/palate. Folate may rescue several impaired signaling mechanisms, including Wnt/β-catenin signaling, epigenetic and oxidative stress mediated defects. Prenatal ethanol exposure produces impaired developmental signaling, oxidative stress and epigenetic regulation, and reports suggest rescue by folic acid treatment.

Normally an essential part of nucleotide synthesis and repair, FA deficiency can have wide ranging consequences. Ethanol consumption interferes with folate absorption; inhibiting its metabolism; increasing excretion of FA; reducing serum and hepatic FA levels; and reducing expression of various folate metabolizing enzymes [63,64]. Due to the involvement of folate in epigenetic events like DNA methylation, ethanol induced folate deficiency was shown to decrease availability of the methyl donor, *S*-adenosyl methionine (SAM) [65]. Hence, FA deficiency may change the epigenetic profile of the developing embryo, leading to changes in gene expression and developmental defects.

Studies in mouse and chick embryos showed that ethanol exposure produces neural tube closure defects like those that result from FA deficiency [31,66]. A clinical study showed a significant reduction in fetal-to-maternal folate ratios in pregnant women after ethanol exposure, indicating reduced folate transfer from the mother to the developing embryos [67]. This data was supported by a significant reduction in folate levels in blood from babies born to alcohol abusing mothers, as compared to control groups. They proposed that antioxidant activity was diminished due to defective maternal-to-fetal folate transfer, causing increased ethanol-induced oxidative stress. A study of mouse embryos suggested that FA has a protective role after ethanol treatment through a microRNA (miRNA) mediated mechanism [68]. Investigators showed elevated levels of specific miRNA (miR10a and miR10b) in ethanol exposed mouse embryos, which could be suppressed by FA supplementation [68]. Using mouse and chick models, Linask and colleagues showed that FA supplementation prevented ethanol induced heart developmental defects [69]. Using zebrafish, the Marrs laboratory showed that FA supplementation significantly rescued body length defect, small eye, and cardiac edema seen in embryos exposed to ethanol alone (Figure 3D,F). Additional studies are needed to determine the effects of ethanol induced FA deficiency on specific neurodevelopmental processes.

#### 2.1.3. Choline

Choline is an essential nutrient, grouped with vitamin B-complex, which plays critical roles as a precursor to the neurotransmitter acetylcholine and various essential cell membrane constituents, including phosphatidylcholine and sphingomyelin. Choline is also thought to influence DNA methylation by serving as a methyl donor and thus regulating gene expression. Like folate, choline is necessary during fetal development, and choline deficiencies lead to severe neural tube defects and CNS dysfunction [70]. Choline supplementation in animal models throughout pregnancy was shown to provide protective and beneficial effects on CNS and behavioral development, and these studies showed that choline supplementation increased neuronal proliferation and differentiation, especially in hippocampus and septum.

Being an essential nutrient, choline deficiency leads to a wide spectrum of defects. Ethanol-induced deficiency of choline may also be responsible for several behavioral deficits. Its deficiency may reduce acetylcholine production and produce long-term learning and memory deficits. Choline deficiency may also reduce DNA methylation, changing epigenetic events that lead to a wide spectrum of defects.

Initial experiments showed that choline supplementation could significantly attenuate severity of ethanol-induced behavioral defects in a FASD model, but not morphological defects [71]. Then, these investigators dissected specific behavioral parameters, including spontaneous alternation, spatial learning and working memory. These studies tested for hippocampal-based learning and memory. Spontaneous alternation, which tests for spatial memory measuring natural exploratory and foraging behavior using the T-maze setup, was significantly affected in ethanol treated offspring and was partially rescued by choline supplementation [72]. The effect of ethanol on spatial and working memory was not as severe, but these defects were considerably improved following choline supplementation. This effect could result from the alteration in ratios of specific cholinergic receptors after ethanol treatment, which could be rescued by choline [73]. To further examine the mechanism underlying choline rescue, Otero and colleagues studied changes in average tissue DNA methylation after ethanol and choline treatments, and compared these to untreated controls. Ethanol treatment caused global DNA hypermethylation in prefrontal cortex and hippocampal brain regions, which was reduced significantly by choline supplementation [74]. Additional research is needed to identify mechanisms underlying choline rescue of specific behavioral parameters and DNA methylation.

Prenatal and postnatal choline intake was reported to be beneficial for enhancing cognitive functions and was shown to change CNS structure and function by increasing neuronal proliferation and differentiation. It is an excellent candidate for preventive and therapeutic treatment to alleviate behavioral and other defects due to prenatal ethanol exposure. However, effects of ethanol on choline uptake and metabolism are not known. Additional research is needed to determine whether choline deficiency is underlying ethanol-induced birth defects and whether choline supplement can protect or restore brain development or function.

#### 2.1.4. Vitamin E

**Figure 4:**
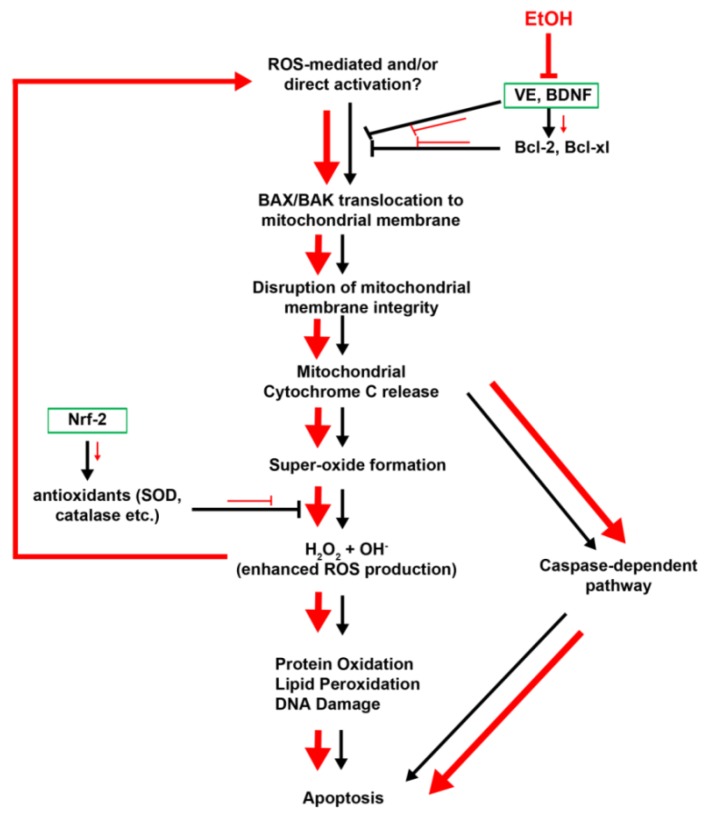
Proposed mechanism for ethanol-induced oxidative stress-mediated cell death. Ethanol can act as an oxidant by inducing the production of reactive oxidative species (ROS) during ethanol degradation. ROS can induce translocation of pro-apoptotic proteins, BAX/BAK to the mitochondrial membrane leading to disruption of membrane integrity. The resulting cytochrome *c* release induces caspase cascade, leading to apoptosis. Cytochrome *c* release also causes increased superoxide formation and enhanced ROS production. This leads to protein oxidation, lipid peroxidation and DNA damage, which can enhance apoptosis induction pathways. Vitamin E and neurotrophic factors such as BDNF and NT3 are known to inhibit BAX/BAK translocation and apoptosis. Anti-apoptotic proteins such as Bcl-2 and Bcl-xl are also affected by to reduced vitamin E (VE) and BDNF. Ethanol reduces vitamin E and neurotrophic factors, which in turn affect Bcl-2 and Bcl-xl. Together, ethanol promotes BAX/BAK translocation to mitochondrial membrane, leading to apoptosis. Key genes like Nrf-2 regulate antioxidant enzymes, including superoxide dismutase and catalase. This is reduced in ethanol-treated cells. Black lines/arrows indicate normal pathway for cell death, and red lines/arrows indicate ethanol-induced changes, increase or decrease. Green boxes indicate therapeutic targets that have been tested for rescue of oxidative-stress mediated cell death.

Vitamin E (VE), α-tocopherol, is a powerful antioxidant and is known to offer protection from ethanol induced neurotoxicity [75,76,77]. During pregnancy, VE deficiency causes several developmental abnormalities and behavioral deficits, which may cause oxidative stress of the rapidly growing embryo [78]. Among the many mechanisms thought to contribute to the etiology of FASD, ethanol induced apoptosis caused by reactive oxygen species (ROS) is well supported and studied. Natural VE can protect against ethanol-induced neurotoxicity in a rat hippocampal cell culture FAS model by preventing lipid peroxidation and preserving membrane integrity, perhaps by scavenging damaging free radicals. Experiments by Heaton *et al.* [79] showed that VE supplementation significantly stimulated secretion of neurotrophic factors such as BDNF, NT-3 and upregulation of anti-apoptotic molecules including Bcl-2, Bcl-xl and pAkt (Figure 4). These effects could promote cell survival and reduce toxic ROS [79].

VE supplementation consistently showed a reduction in ethanol-induced cell loss within certain brain structures [75]. However, these VE treatments failed to rescue behavioral deficits. Ethanol-induced VE deficiency and VE enhanced neuronal survival are not well understood.

### 2.2. Ethanol-Induced Oxidative Stress: ROS Generation and Cell Death

Ethanol-induced neurotoxicity was primarily attributed to ROS-induced cellular damage. Together with nutrient deficiencies and other effects, ethanol can increase the production of ROS, which are coupled with decreased levels of antioxidant defenses, redox imbalance and oxidative damage to lipids, proteins, and DNA. This oxidative stress can contribute, in part, to the neurodegeneration effects observed in FASD. This was extensively reviewed by Brocardo *et al.* [80].

Studies on cerebellar granule cell cultures were used to dissect the pathways upstream and downstream of ethanol-induced ROS production (Figure 4). Ethanol-exposure induced pro-apoptotic protein upregulation and decreased anti-apoptotic protein expression, mitochondrial release of cytochrome *c*, and, finally, triggering cell death (Figure 4). Some studies support that ethanol-induced neurotoxicity also interferes with expression of neurotrophic factors (e.g., BDNF, NT-3 [79]), and reduced growth factor expression induce neuronal cell death. This interference in neurotrophic factor expression can be a result of reduced vitamin E, which is involved in stimulation neurotrophic and anti-apoptotic factors secretion (see above), a direct reduction in expression by ethanol, or a secondary pathway yet to be elucidated. Numerous exogenous antioxidants, including superoxide dismutase (SOD), catalase, VE, *N*-acetylcysteine and lipoic acid, significantly decrease apoptosis in ethanol-exposed embryos [81,82,83]. Many nutritional antioxidants, including various vitamins (C, E), folate and flavonoids showed protective effects (Figure 4) by reducing ethanol-induced oxidative stress [84]. Activation of key antioxidant proteins, such as Nrf-2 (Figure 4) by chemical gene activators like 3H-1,2 dithiole-3-thione (D3T) were also tested for their neuroprotective effects on ethanol exposed embryos [85]. Providing neurotrophic support, including BDNF, VE and PPAR-γ agonists [86], were studied as viable therapeutic targets, and antioxidant compounds also provide a potential therapeutic approach, which can be tested in ethanol-treated animal models. In summary, antioxidants may be effective, alone or in combination with other therapeutic agents to mitigate neural defects and behavioral deficits observed in individuals affected with FASD.

### 2.3. Cell Death

**Figure 5:**
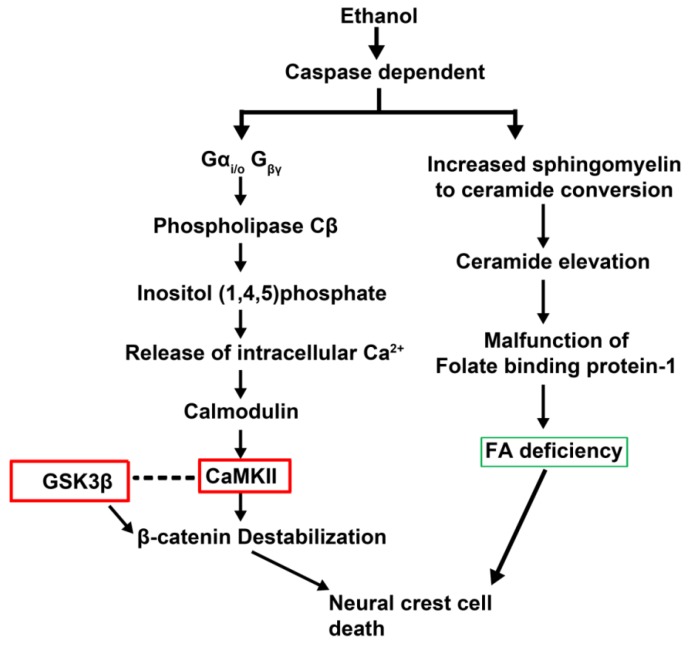
Proposed mechanisms of ethanol-induced cranial neural crest cell death. Ethanol exposure can induce the caspase cascade, which leads to apoptosis of the cranial neural crest cells (CNCCs) via two known mechanisms. Ethanol can activate G-protein coupled receptors, activating phospholipase C, which breaks down phosphatidylinositol-bisphosphate to IP_3_ and DAG. IP_3_ mediated release of intracellular calcium stores leads to increased Ca^+2^ dependent kinase activation. Calmodulin and CamKII activation leads to phophorylation and proteolysis of β-catenin, which affects gene expression and CNCC apoptosis. Reducing β-catenin degradation is a potential therapeutic target, and kinase inhibitors for CamKII and GSK3β (canonical Wnt inhibitor) have been tested (red boxes). Ethanol-induced production of ceramide from sphingomyelin is another potential mechanism that leads to the malfunction of folbp-1, folate binding protein-1, and contributing to FA deficiency. This can lead to increased CNCC death. Supplementation with FA can potentially rescue CNCC death (green box). Based on studies from Smith and colleagues [80,81,82,84]. Dotted line indicates possible interaction between canonical and noncanonical Wnt signaling pathways.

In addition to ROS-mediated cell death, other studies showed specific pathways participate in apoptosis of CNCCs (Figure 5). A study showed CNCC cell death following ethanol exposure occurred via a ceramide-induced, caspase-3-mediated pathway [87]. These investigators showed an increased conversion of sphingomyelin (ceramide precursor molecule) to ceramide in ethanol-treated CNCC cultures. Their results suggest that defects in lipid metabolism, like sphingomyelin. The investigators propose that this can lead to reduced sphingomyelin dependent lipid-rafts in the neural-tube associated cells leading to folate-binding protein 1 (folbp-1) malfunction and resulting in folate deficiency. The investigators also proposed that ethanol treatment reduced levels of one carbon metabolism pathway intermediates, such as SAM, phosphatidylcholine and sphingomyelin. One carbon metabolism functions that heavily rely on folate and vitamin B complex were affected. However, additional detailed examination is required to determine the players of this pathway.

Investigators proposed calcium-mediated β-catenin destabilization as an underlying mechanism for CNCC apoptosis (Figure 5) [88,89] Studies on developing chick embryos suggest ethanol exposure induced activation of the GPCR, G_αi/o_, and elevated IP_3_ levels via activation of Phospholipase Cβ. IP_3_-mediated release of calcium stores could directly affect non-canonical Wnt/Ca^2+^ signaling pathway. Evidence showed destabilization of β-catenin, reduction in β-catenin-mediated transcriptional activation and initiation of apoptosis after ethanol exposure in the neural folds. The hypothesis that ethanol exposure interferes with β-catenin function and induces apoptosis is consistent with the previously proposed role of β-catenin in CNCC survival. These investigators also suggest that ethanol may act through the downstream effectors of the non-canonical Wnt pathway, including calcium mediated kinases and proteases like CaMKII, PKC and calcineurin-NFAT. These enzymes could induce ubiquitination and proteolytic degradation of β-catenin [90]. 

A small molecule screen showed that calmodulin inhibitors significantly enhanced CNCC survival in ethanol-treated embryos [91]. These studies showed that CaMKII activation leads to alcohol neurotoxicity. CaM kinases are targets of activated calmodulin, and inhibitors of CaM kinases were able to rescue CNCC death. CaMKII activation may cause ubiquitination and proteolytic destruction of nuclear β-catenin, which negatively feeds back on cell survival gene expression in CNCCs. β-Catenin degradation can result from CaMKII activation directly or by disrupting the critical balance between non-canonical Wnt and canonical Wnt signaling. This disruption could help explain ethanol-induced effects on β-catenin stability, which affects canonical Wnt functions like cell survival, proliferation and migration. As a potential therapeutic tool for canonical Wnt signaling, GSK3β small molecule inhibitors, which stabilize nuclear β-catenin, could be tested for their ability to prevent CNCC apoptosis and preserve CNCC development during embryogenesis.

In summary, there are several potential targets for preserving CNCC survival. To target β-catenin ubiquitination and proteolysis, small molecule inhibitors of GSK3β and CaMKII may provide potential therapeutics for testing [92].

### 2.4. Epigenetics

Epigenetics are heritable, and potentially reversible, changes in the functionally diverse cells of a multicellular organism, which governs the compaction and folding of chromatin structure mainly by covalent modifications on DNA and histones. Epigenetic mechanisms regulate gene expression at the level of transcription by modulating the accessibility of gene promoter regions to the transcriptional machinery.

The chromatin modifications include DNA methylation, histone posttranslational modifications (methylation, phosphorylation, acetylation and others). The interplay between DNA methylation, histone modifications and a variety of chromatin modulating complexes regulates chromatin folding, and thus, gene expression. Epigenetic modifications are produced by specific enzymes, including DNA methyltransferases (DNMTs), histone methyltransferases (HMTs), histone acetyltransferases and deacetylases (HATs and HDACs respectively).

Several nutritional factors regulate the epigenetic profile, including dietary supplements like FA, choline, vitamin B12 and other nutrients. Studies suggest that environmental factors, like alcohol and drugs, have a negative impact on epigenetic modifications [93]. Epigenetic modifications induced by drug exposure *in utero* were associated with long-lasting behavioral phenotypes, including hyperactivity and psychiatric disorders in children [94].

Ethanol interferes with components of the one carbon metabolism pathway, including folate, choline (a derivative of homocysteine) and SAM (methyl donor). Disrupting one carbon metabolism can lead epigenetic alterations and global changes in epigenetic profiles. Epigenetic changes induced by embryonic ethanol exposure were recently reviewed [95,96]. Pregnant mice treated with ethanol showed significant embryo hypomethylation [97]. Classic experiments also showed significantly reduced offspring growth due to preconceptional (60 days prior through conception) maternal or paternal alcohol exposure, indicating that directly exposing offspring to alcohol is not necessary to induce developmental abnormalities. Thus, epigenetic changes in the germ line could be an additional contributing factor in FASD.

Mechanistic studies in mouse models showed that prenatal ethanol exposure increased embryo DNA methylation. In these studies, investigators used epigenetically sensitive strains of mice expressing reporter yellow pigment (Agouti viable yellow, Avy mice). Neonates born after ethanol exposure showed decreased expression of pigment, indicating suppression of pigment gene expression due to DNA hypermethylation [98]. However, whether these ethanol induced epigenetic changes were site-specific was not studied. Altered DNMT expression levels were seen in sperm cells of male rats exposed to ethanol [99]. Ethanol-induced alterations DNA methylation profile during early neurulation in developing mouse embryos and neural stem cell cultures were also observed. High-throughput methyl DNA microarray analysis identified specific DNA methylation changes associated with dysregulation of neural stem cell migration and differentiation program after ethanol exposure [100]. These experiments also identified global epigenetic changes in and around promoter regions due to ethanol exposure, which correlated to growth retardation in developing embryos [101]. This suggests that alcohol exposure prevented normal DNA methylation progression, a critical sequence of events during differentiation of neural cells [100]. The DNA methylation program during development from blastocysts, during gastrulation, and through neurulation utilize 5methylcytosine (5mC) and 5hydroxylmethylcytosine (5hmC) changes in DNA, which are spatiotemporally regulated [93]. This was also recently documented in the developing nervous system [102]. Alcohol affects on spatiotemporal progression of the DNA methylation occurred in the neural tube [103] and hippocampus [102]. Inhibiting DNA methylation produced similar growth retardation effects in the embryos during neurulation [103].

In addition to the DNA methylation, studies on neural stem cells showed that histone modifications including tri-methylated histone 3 on lysine 27 (3me-H3K27), di-methylated histone 3 on lysine 4 2me-H3K4, and acetylated H4 (Ac-H4) are closely tied to the migration of neural stem cells and their differentiation [104]. Recent experiments on mouse embryos showed that ethanol exposure enhanced transcriptional activation of G9a, a histone methyltransferase (HMT), which produces the H3 modifications 2me-H3K9 and 2me-H3K27. These modifications led to H3 degradation and neurodegeneration. Pharmacological inhibition of G9a activity before or during ethanol exposure could restore H3 levels and rescue neural cell survival [105]. In another study, choline supplementation during ethanol treatment could rescue expression levels for DNMTs and histone-modifying enzymes [106]. Choline deficiency was previously shown to alter G9a activity. Quantitative PCR studies showed rescue of G9a and Setdb1 (HMTs) expression by choline supplementation. Choline supplementation could also rescue DNMT expression levels in ethanol-treated mouse embryos.

One potential mechanism underlying ethanol-induced disruption of one-carbon metabolism could be a direct effect on epigenetic regulators. Another possibility can involve ethanol-induced effects on gene expression via intermediate mechanisms, which could include epigenetic modulating factors. Experiments on early zebrafish embryos showed changes in expression levels of specific epigenetic modulators [28]. Changes in the epigenome greatly alter gene expression profiles, which ripples down to various developmental or physiological signaling disruptions. Effects of ethanol on DNA and histone methylation pathways are not clearly understood, but these effects appear to be very central to the ethanol-induced defects as illustrated by the rescue effects of various epigenetic modifiers.

### 2.5. Non-Coding RNA

In recent years, non-protein-coding regions of the genome were shown to play a critical role in regulating normal developmental processes, physiology and disease [107]. Roles for noncoding RNA (ncRNAs) especially micro-RNA (miRNA) were studied as regulators of various biological processes, including cell cycle, fate specification, differentiation, and cell death. These miRNAs genes are transcribed by RNA polymerase II, processed in the nucleus and assembled into RNA-induced silencing complexes (RISC) in the cytoplasm. These complexes inhibit gene expression by binding to mRNAs and inhibiting translation. Various diseases were linked to changes in miRNA expression including cancer, neurodegenerative and metabolic diseases. Although involvement of miRNA in FAS model was predicted [108], only a few studies have focused on specific miRNA dysregulation due to ethanol exposure and its role in manifestation of FASD.

Initial cell culture studies examined specific neuronal miRNAs, showing that some miRNA expression levels changed after ethanol treatment. Ethanol caused some miRNAs to be highly expressed and others to be downregulated [109]. Ethanol-induced upregulation of miRNA-335, -21, -9 and -153 could disrupt neuronal specification and differentiation process. Jag-1, a notch ligand is activated by coordinated suppression of miRNA-335, -21 and -153. A proneural gene involved in neuronal maturation, Evalv2, was upregulated by suppression of miR-335, -153 and -9. These findings suggest that altered miRNA gene expression induced by prenatal ethanol is a mechanism for neuronal birth defects.

Wang *et al.* [68] showed significant upregulation of miR-10a and -10b and other miRs in ethanol treated mouse embryos. miR-10a and 10b are located adjacent to a *Hox* gene cluster and target *Hoxa1* expression, inhibiting its translation in ethanol treated embryos. These investigators also found that inhibition over *Hoxa1* translation was rescued by FA supplementation in a dose-dependent fashion, without altering *Hoxa1* mRNA levels. This strongly suggests a post-transcriptional or translational mechanism (like miRNA-mediated) of gene expression regulation. Microarray analysis on zebrafish embryos treated with high and low doses of ethanol showed effects on a several miRNAs [110]. Different sets of miRNAs were affected by different doses of ethanol in a complex pattern. These results also showed upregulation of miR-9, which contradicted the earlier study. Together, these findings suggest that miRNA genes were differentially affected by ethanol depending on concentration and the tissue type. Predicted targets for many of the affected miRNA genes are neural developmental signaling mechanisms. Additional research is needed to understand the complex mechanisms underlying ethanol-induced changes in miRNA expression and to identify subtypes of miRNAs that are particularly sensitive to ethanol exposure

## 3. Conclusions

Molecular mechanisms underlying ethanol-induced defects require close examination to identify specific targets of ethanol toxicity during development. These primary targets may lead to various cell-specific and tissue-specific changes. For instance, early ethanol exposure caused CNCC apoptosis, and CNCCs are involved in multiple organ cell specification and differentiation events. Interrupting cell signaling and tissue interactions could lead to defective cellular structures, gene expression, tissue architecture and organ function. However, most studies focusing on specific defects in a developing or mature organ fail to examine lineages of precursor cell types and their defects. In addition to molecular mechanisms, genetic, nutritional and environmental factors may alter the severity of ethanol-induced defects in the developing embryo. 

Mechanisms outlined above are not independent of each other, suggesting that multiple ethanol targets produce a complex interplay between pathways, which cascade down to a myriad of changes at DNA, RNA and protein levels. If true, then combination of therapeutic agents may be necessary to mitigate the FASD deficits. However, there may be nutritional or developmental pathways that are centralized and may provide key support for the developing embryo. New studies are needed to define and maximize rescue effects to prevent neural damage.

Many of the proposed interventions mentioned above, like nutritional supplements, are preventive measures that may reduce the severity of ethanol-induced birth defects. Advances in stem cell-based and other regenerative therapies could bring therapeutic benefits for neural defects associated with ethanol exposure. A recent study used neural stem cells to rescue neuronal cell death induced by prenatal alcohol exposure and showed potential therapeutic efficacy for memory and social recognition deficits in neonatal rats [111]. Using atellocollagen (purified collagen) to deliver the neural stem cells, these investigators provided a physical scaffold for the proliferating cells, which may potentiate their migration and reduce immune rejection. In addition to evaluating cell based therapies, these stem cell approaches can be used to evaluate abnormal cellular mechanisms associated with FASD. Medical treatments using fetal brain-derived NSCs or embryonic stem cells have important ethical issues, which must be fully explored. However, the potential benefit of advanced therapeutics warrants our efforts to help patients through additional FASD research, which may lead to targeted interventions that attenuate the numerous birth defects associated with prenatal alcohol exposure.
